# Dietary Factors May Delay Tolerance Acquisition in Food Protein-Induced Allergic Proctocolitis

**DOI:** 10.3390/nu15020425

**Published:** 2023-01-13

**Authors:** Gavriela Feketea, John Lakoumentas, George N. Konstantinou, Nikolaos Douladiris, Nikolaos G. Papadopoulos, Maria Petrodimopoulou, Ioannis Tasios, Mina Valianatou, Vasiliki Vourga, Emilia Vassilopoulou

**Affiliations:** 1 Department of Hematology, “luliu Hatieganu” University of Medicine and Pharmacy, 400337 Cluj-Napoca, Romania; 2 “Karamandaneio” Children’s Hospital of Patra, 26331 Patra, Greece; 3 Department of Nutritional Sciences and Dietetics, International Hellenic University, 57400 Thessaloniki, Greece; 4 Department of Allergy and Clinical Immunology, 424 General Military Training Hospital, 56429 Thessaloniki, Greece; 5 Allergy Department, 2nd Pediatric Clinic, National and Kapodistrian University of Athens, 11528 Athens, Greece

**Keywords:** cheese, food allergy, food protein induced allergic proctocolitis, grilled food, meat, Mediterranean diet, olive oil, ready-to-eat food, sugars

## Abstract

Background: Dietary and environmental factors may influence tolerance acquisition in food protein-induced allergic proctocolitis (FPIAP). This retrospective observational study explored the role of maternal diet during pregnancy and breastfeeding in tolerance acquisition in infantile FPIAP. Methods: Breastfed infants with FPIAP from six diverse regions in Greece were divided into two groups, based on development of tolerance to the trigger food: Group A (n = 43), before, and Group B (n = 53), after, the 6th month of age. Maternal diet during pregnancy and breastfeeding was elicited using the Mediterranean Diet Score Questionnaire and the Mediterranean Oriented Culture Specific Semi-Quantitative Food Frequency Questionnaire. Results: Mean age at diagnosis of FPIAP (1.5 months) and weaning (5.5 months) were the same in both groups. The main trigger was cow’s milk. Group A received infant milk formula earlier than Group B. Group B had a higher incidence of asthma/wheeze, siblings with milk allergy, maternal smoking and rural residence. On multivariate analysis, earlier resolution of FPIAP was associated with higher maternal education and with salt intake and consumption of goat/sheep cheese during pregnancy and olive oil during breastfeeding. Consumption of multivitamins during pregnancy and meat, winter fruits, green vegetables, butter, salt, “ready-to-eat” meals and pastries during breastfeeding were correlated with longer duration of symptoms. Conclusions: Mothers of children with FPIAP to cow’s milk protein can be advised to eat more yogurt, cheese and olive oil during subsequent pregnancies, and avoid multivitamins, grilled food, “ready-to-eat” meals, pastries, meat and alcohol during breastfeeding, to reduce the duration of FPIAP presenting in future infants.

## 1. Introduction

Food protein-induced allergic proctocolitis (FPIAP), formerly known as allergic or eosinophilic proctocolitis or “protein intolerance”, is a common problem in young infants [1,2,3]. A cumulative incidence of 17% has recently been reported in an unselected population [4]. Reported rates of FPIAP are higher in Greece and Brazil, countries with low rates of food allergy (FA) in general [5]. FPIAP often presents as rectal bleeding in an otherwise healthy infant, although some may present significant irritability and diarrhea [6,7]. It usually begins in the first weeks of life and, in most cases, resolves by late infancy [8].

FPIAP is characterized by inflammation of the distal colon in response to one or more food proteins, with no evidence of immunoglobulin E (IgE) involvement [9]. Milk, egg and soy are the foods most commonly implicated [10,11]. The association of symptoms with food protein antigens is confirmed by objective improvement following withdrawal of the suspected food antigen and, in some cases, recurrence following subsequent oral challenge [2]. In a case series in a Greek population, the most common trigger in breastfed infants was cow’s milk (83.6%), followed by egg (7.3%), wheat (6.4%) and beef (6.4%). Interestingly, a possible beneficial effect of maternal consumption of certain foods during pregnancy and breastfeeding in preventing FPIAP development (including whole grain products, fish, shellfish and fruits) was suggested [11].

When FPIAP occurs, permanent resolution of symptoms occurs and tolerance typically develops during the first year of life [2,12] and the trigger food can then be reintroduced in the mother’s and/or the infant’s diet. The dietary and environmental factors that might affect tolerance acquisition have not been extensively studied. The purpose of this study was to investigate the role of potential nutritional and environmental risk factors during pregnancy and lactation that might delay tolerance acquisition in FPIAP in infants.

## 2. Methods

### 2.1. Participants

A retrospective, observational, multicenter study was conducted between May 2018 and November 2020 on 96 mothers and their breastfed infants with a diagnosis of FPIAP confirmed by a pediatric allergist, recruited from 6 regions in Greece Athens, Thessaloniki, Amaliada, Volos, Kavala and Kozani) with different environmental characteristics, as previously described [11].

The study was approved by the Hospital Ethics and Scientific Committee of the Amaliada General Hospital of Ilia and the Medical Association of Thessaloniki on behalf of all the study centers (Study IDs: 53/2021 and 4170/2018) and was conducted in accordance with the code of Ethics of the World Medical Association (Declaration of Helsinki). The participating mothers provided their written consent after being informed of the scope and procedures of the study.

The participants were divided into two subgroups, based on the time of tolerance acquisition to the trigger food causing FPIAP symptoms: Group A consisted of mothers and their infants in whom resolution of FPIAP was observed before the 6th month, and Group B consisted of mothers and their infants in whom resolution was observed after the 6th month of age.

### 2.2. Clinical Data

The diagnosis of FPIAP was made in each case by a pediatric allergist, based on a history of scant bright red rectal bleeding with mucus in an otherwise healthy infant that resolved within two weeks with a strict maternal elimination diet and recurred after reintroduction in the maternal diet of the culprit food. Infections and other causes of rectal bleeding, such as volvulus, intussusception, necrotizing enterocolitis and Hirschsprung’s disease were excluded. The presenting symptoms, the infant’s age at onset and at development of tolerance and at weaning, physical examination findings, stool examination results and food(s) implicated in FPIAP were retrieved from the medical records.

### 2.3. Questionnaires

Information regarding the family’s demographic characteristics, including place of residence, educational level, occupation, smoking habits, were collected. An allergy-focused questionnaire containing data about self-reported allergic symptoms, laboratory testing and physician-diagnosed allergic disease has been assessed. Moreover, two food frequency questionnaires on maternal eating habits during pregnancy and breastfeeding were administered by personal interview.

#### Food Frequency Questionnaires

(a) The Mediterranean Diet Score Questionnaire (MedDiet score), which estimates adherence to the Mediterranean Diet (MedDiet), recording consumption of the 11 main components (non-refined cereals, fruit, vegetables, potatoes, legumes, olive oil, fish, red meat, poultry, full-fat dairy products, and alcohol). The resultant MedDiet score is categorized: 0–13: no adherence; 14–27: insufficient; 28–41: satisfactory; 42–55: very good adherence [13].
(b) The Mediterranean Oriented Culture Specific Semi-Quantitative Food Frequency Questionnaire, which includes 221 foods, subdivided into 22 food sections: (1) white grain products, including rice and potato; (2) whole wheat grain products; (3) breads/pastries; (4) stews; (5) pulses; (6) raw and cooked vegetable salads; (7) fruit and homemade juices; (8) nuts; (9) milk/dairy products; (10) meat/traditional meat dishes; (11) red meat products; (12) white meat products; (13) eggs; (14) fish/seafood; (15) fats/spreads (including olive oil); (16) traditional dips/sauces/dressings; (17) sugar/sweet preserves/confectionary; (18) “ready-to-eat” foods, including restaurant food in, delivery food and prepacked cooked food; (19) chips/salty puffed snacks; (20) herbal infusions/teas; (21) soft drinks/nonalcoholic beverages; (22) alcoholic drinks. Four additional questions concern (23) addition of extra salt, (24) use of non-stick casseroles/grilled food, (25) traditionally cooked Mediterranean homemade food, and (26) food supplements.

The above components were scored as: 1 = never, or less than once per month, 2 = 1–3 times per month, 3 = once per week, 4 = 2–4 times per week, 5 = 5–6 times per week, 6 = once per day, 7 = 2–3 times per day, 8 = 4–5 times per day and 9 = 6 or more times per day [14].

### 2.4. Statistical Analysis

A mixed dataset was available for the analysis, which contained nominal categorical variables (no/yes), ordinal categorical variables (range 0–5) and scale variables. The ordinal categorical variables were treated as scale variables in the analysis. Scale variables were all non-parametric, according to the Shapiro–Wilk test for composite normality, and were described as median (Q1–Q3). All nominal categorical variables were described as count and % relative frequency. Where aggregation of variables was required, nominal categorical variables were merged with the “ANY” operator (for example, if ANY of the siblings presented milk allergy, siblings’ milk allergy was set to YES, otherwise to NO), and scale variables were aggregated with average. Inferential statistical analysis was performed via hypothesis testing. To associate each independent variable with the categorical 2-leveled variable of the age of tolerance in months: 0–6 vs. 6–48, the Wilcoxon’s rank sum test (for scale independent variables) and Pearson’s χ^2^ test of independence (for categorical independent variables) were applied. *p*-values equal to or less than 0.05 indicated statistical significance. All statistical hypothesis tests were considered as two-sided.

### 2.5. Machine Learning Analysis

Machine (supervised) learning methods were utilized to predict the age of tolerance, given a set of independent variables as predictors [13]. The target of the prediction task was the discrete 2-leveled variable: [0–6) vs. [6–48) in months. The classification tree ensemble XGBoost method, with the game theoretical SHAP metric for interpretability, were utilized for prediction [14,15]. The evaluation involved a 6-fold cross validation, with the use of the metrics of accuracy, sensitivity, specificity, F1-score and the receiver operating characteristic (ROC) curve area. ROC curve analysis was used for visualization, along with the SHAP metric that provides an explanation diagram for binomial classification and also corresponds to the variable importance diagram for classification. Hyperparameter tuning was applied with grid exploration in order to increase performance. Prior to modeling, all independent variables were imputed with random forest imputing for mixed data [16] and they were min–max normalized (all predictors, either scale or categorical, were brought within the [0,1] interval). The methods were implemented and visualized with the R language for statistical computing and with the assistance of the RStudio IDE, two projects that are well known in the data analytics community and are also open source (R: 4.1.2 2021-11-01, RStudio 2021.09.1+372). The libraries used, apart from base, were xgboost, SHAPforxgboost, MLmetrics and ROCR.

## 3. Results

Of the 96 infants with a diagnosis of FPIAP, 43 (44.8%) showed resolution of FPIAP before the 6th month (Group A) and 53 (55.2%) showed resolution after the 6th month of age (Group B). Blood and mucus in the stools were the main symptoms in all infants (*p* > 0.05) and three infants in Group B also presented anemia. The mean age at diagnosis of FPIAP was 1.5 (1–3) months in both groups. Τhe median age at acquisition of tolerance was 12 (11–14) months, with 44.79% of cases achieving tolerance by the age of 6 months, 23.95% between 6 and 12 months, 20.83% between 12 and 18 months and only 10.41% after the age of 18 months. Group A infants acquired tolerance at 4 (2.75–5) months and Group B at 12 (7–16) months (maximum age at tolerance was 48 months). Table 1 shows the characteristics of the study population.

The mean time of food introduction was slightly, but not significantly, earlier in Group B [5.5 (5–6) months] than in Group A, [6 (5.5–6) months] (*p* = 0.41).

The main trigger food of FPIAP was cow’s milk, as 42/43 infants in Group A and 50/53 infants in Group B presented symptoms during the breastfeeding period after cow’s milk consumption by the mother. Other trigger foods for both groups were egg (one in Group A, seven in Group B), and beef (one in Group A and six in Group B). For Group B wheat (11.32%), corn (3.77%), soya (5.66%), lamb, peanut, fish, pear and grape (1.89%) also induced FPIAP symptoms (Table S1 Supplementary Material)

A dairy avoidance diet was followed after the FPIAP diagnosis by 29/43 Group A mothers and 31/53 Group B mothers who continued breastfeeding. In addition, 11/53 of Group B mothers followed another food avoidance diet to continue breastfeeding. Group A infants, in addition to breastfeeding, started an infant milk formula, specific for age, at an earlier age [0 (0–3) months] than Group B infants [3 (1–5.5) months] (*p* < 0.001); 26.42% of Group B and 4.65% of Group A infants were prescribed an elemental formula (*p* = 0.01). No other significant differences were reported in the use of other formulas, such as extensively hydrolyzed formula (eHF), amino acid, hypoallergenic formula (HA) and probiotics, or in the duration of exclusive breastfeeding or time of introduction of fresh cow’s milk in the diet (*p* > 0.05) (Table S2 supplementary material).

Regarding the individual history of other allergies at the age of data collection, no significant differences were observed between Group A and Group B infants, apart from wheezing in some Group B infants (15.09% of Group B, *p* < 0.02) (Table S3 supplementary material). A trend towards higher eczema presentation was reported in Group B infants, but the difference was marginally significant (*p* = 0.05).

Group B infants more often had a sibling with a history of cow’s milk allergy (9/53, 16.98%) than Group A infants (1/43, 2.33%) (*p* = 0.04). Otherwise, no significant differences were detected in the family allergy history (Table 2).

Smoking was more frequent among the mothers of Group B infants (*p* = 0.04), who had a lower level of education (*p* < 0.001) and were more likely to live in a rural area (Table 2).

During pregnancy, the mothers of the infants in the two groups reported similar, moderate, adherence to the MedDiet (*p* > 0.05). Group A mothers more frequently consumed olive oil (*p* < 0.001), whole fat yogurt (*p* = 0.031), white meat (*p* = 0.02), goat/sheep cheese (*p* = 0.007) and table salt (*p* = 0.018) than Group B mothers. Group B mothers reported a relatively higher intake of butter (*p* = 0.007), seed oil (*p* = 0.02), margarine (*p* = 0.004), alcohol (beer) (*p* = 0.03), grilled food (*p* = 0.015), “ready-to-eat” meals outside the house (*p* = 0.04) and pastries (*p* = 0.04). In addition, Group B mothers used multivitamins more frequently than Group A mothers (43.4% vs 18.6%, *p* = 0.01). The eating habits during pregnancy of the mothers of the infants with FPIAP are shown in Table 3.

During the breastfeeding period, the Group A mothers more frequently avoided nuts (*p* < 0.001), shellfish (*p* = 0.01) and pork (*p* = 0.002) without relevant guidance by the physician (Table 4). The Group A mothers consumed more olive oil (*p* < 0.001) and goat/sheep cheese (*p* < 0.039), but less butter (*p* = 0.008), margarine (*p* = 0.001) and mayonnaise (*p* = 0.001). They also recorded a lower intake of grilled food (*p* = 0.01), green vegetables (*p* = 0.04), boiled vegetables (*p* = 0.03), olives (*p* = 0.03), fermented vegetables (*p* = 0.003), oranges and winter fruit (*p* = 0.006), deli meat (*p* = 0.008), white (*p* = 0.02) and red meat (*p* = 0.03), pastries (*p* = 0.02) and alcohol (*p* = 0.028). No significant differences were found in the avoidance of the food allergens implicated with FPIAP symptoms between the two groups (i.e., milk, egg, beef, corn, soya, lamb, peanut, fish, pear and grape) (Table 4).

### Multivariate Analysis Results

**Figure 1:**
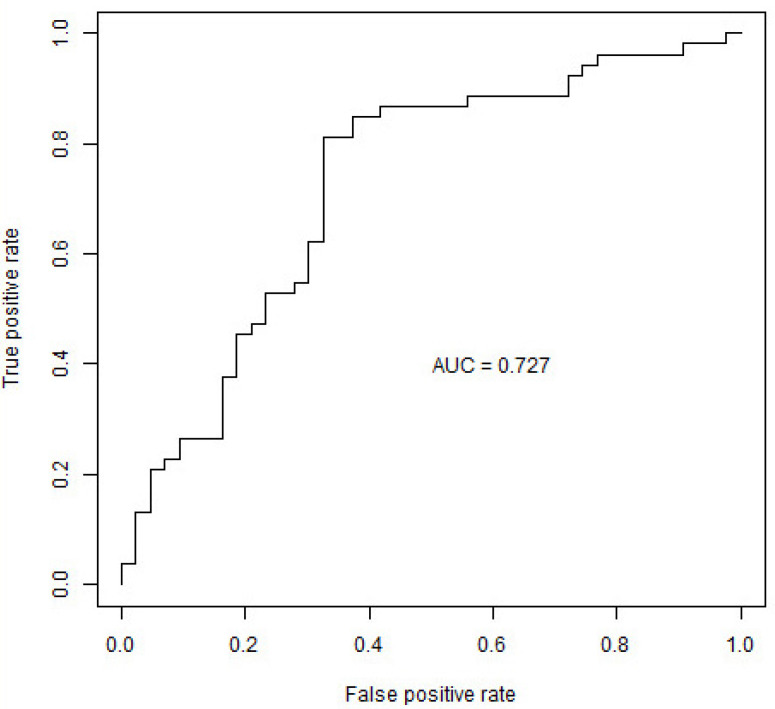
Prediction of the age of tolerance acquisition of infants with food protein-induced allergic proctocolitis (FPIAP): The Receiver Operating Characteristic (ROC) curve of the testing phase of the XGBoost classifier. AUC: Area Under Curve.

Following univariate analysis, the XGBoost classifier, incorporated the 29 features with *p* < 0.05 to predict the time of tolerance acquisition in group A compared with group B (detailed information on multivariate analysis is provided in the supplementary material). There was a success of 100% (96/96) during training, regarding the correct prediction of subjects, with an accuracy of 0.74 (71/96) during testing and ROC area under the curve (AUC) of 0.72 (Figure 1)

**Figure 2:**
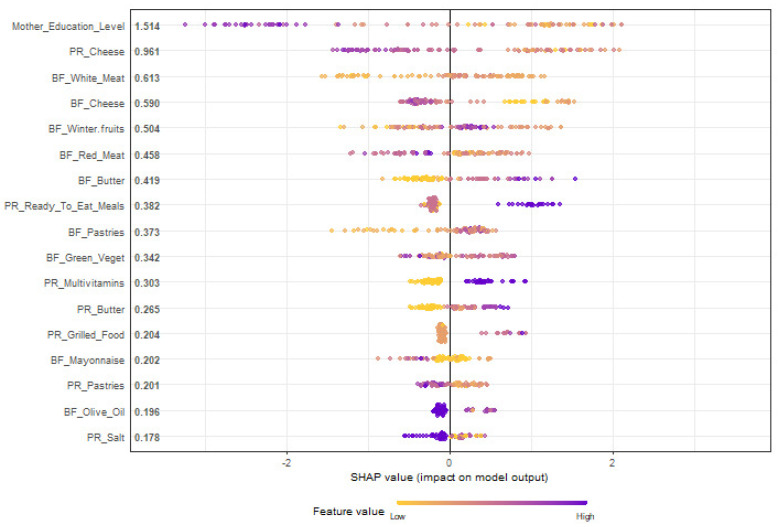
Prediction of the age of tolerance acquisition of infants with food protein-induced allergic proctocolitis (FPIAP): SHAP plot of the training phase of the XGBoost classifier. PR: Pregnancy; BF: Breastfeeding; Green_Veget: Green Vegetables. Predictor values are symbolized with colors from low to high. Using the SHAP diagrams, we evaluate the association, positive or negative, between each predictor and the target class. According to the SHAP diagrams, once the purple-to-blue dots per variable fall mostly on the left side, they favor group A, while once they fall on the right side, they favor group B, confirming the findings from the univariate analysis.

**Figure 3:**
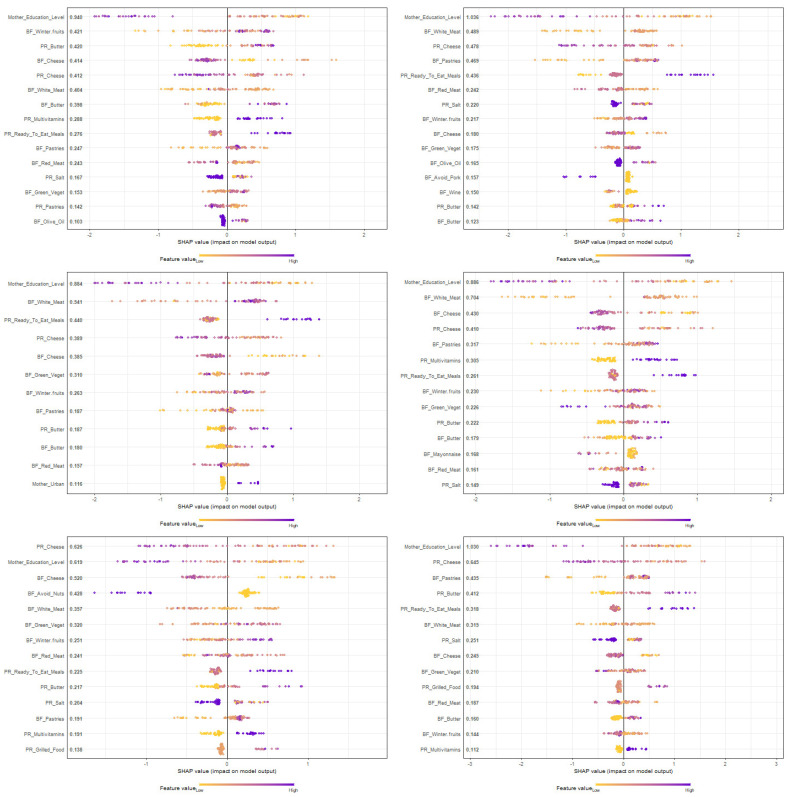
Prediction of the age of tolerance acquisition of infants with food protein-induced allergic proctocolitis (FPIAP): SHAP plot of the testing phase of the XGBoost classifier: one separate plot per fold of the 6-fold cross validation process of testing. PR: Pregnancy; BF: Breastfeeding; Green_Veget: Green Vegetables. Predictor values are symbolized with colors from low to high. Using the SHAP diagrams, we evaluate the association, positive or negative, between each predictor and the target class. According to the SHAP diagrams, once the purple-to-blue dots per variable fall mostly on the left side, they favor group A, while once they fall on the right side, they favor group B, confirming the findings from the univariate analysis.

To interpret the contribution of each predictor individually, the SHAP diagrams were illustrated, both for training (Figure 2) and the 6 folds of testing (Figure 3). The predictors included in a SHAP diagram are only those that achieve a mean SHAP > 0.1.

Among the parameters included in the model, earlier resolution of the FPIAP was always favored by a higher level of maternal education and higher consumption of goat/sheep cheese during pregnancy. Salt intake during pregnancy and olive oil during breastfeeding were correlated with earlier resolution. Conversely, consumption of multivitamins during pregnancy and intake of white and red meat, winter fruits, green vegetables, butter, salt, “ready-to-eat” meals and pastries during breastfeeding were correlated with longer duration of symptoms.

## 4. Discussion

Environmental and nutritional factors during pregnancy and breastfeeding may affect the development of FPIAP [17], as we reported in a previous study [11]. A maternal MedDiet, with large amounts of whole grain products, fish, shellfish, fruits and home-cooked food, has been shown to be associated with a lower risk of FPIAP [12] and other allergies [14], while increased intake of vegetable oils, margarine and “fast foods” appears to be related to a higher risk [12,18]. There has been no previous investigation of nutritional and environmental factors that may affect the development of tolerance in infants with FPIAP. In this first relevant study, we found that consumption of goat/sheep cheese and olive oil during pregnancy and lactation and avoidance of “ready-to-eat” meals and pastries might shorten the period of induction of tolerance.

In our study, the median age of the infants at resolution of FPIAP was 12 (range 11–14) months, with 44.79% of cases achieving tolerance by the age of 6 months and 68.75% by 12 months, while only 10.41% of the infants acquired tolerance later, by the age of 18 months. In contrast, other studies report fewer infants achieving tolerance before the age of 12 months, and some not until 18–24 months [19,20,21,22,23,24,25,26]. The high level of adherence to the MedDiet of the mothers in our study [11] and the distinct Greek nutritional habits could explain these differences, in accordance with the previous suggestion of a beneficial role of the MedDiet [27].

We evaluated several factors that have been proposed to be related to the achievement of tolerance in FPIAP in two groups of infants, taking 6 months of age as a cut-off point for early tolerance. Current infant feeding guidelines, including those of WHO and UNICEF, recommend the introduction of complementary foods at 6 months of age, with continued breastfeeding for up to 2 years of age [28,29,30]. In breastfed infants, differences in breast milk composition, such as the levels of cytokines or the presence of immunoglobulins, could promote the development of tolerance to antigens present in breast milk [31,32,33]. The study of Cetinkaya and colleagues supported the theory that early introduction of complementary feeding accelerates tolerance in FPAIP [20]. In light of these findings, a timely decision should be taken about the initiation of complementary foods in order to avoid unnecessarily restrictive diets.

The most common triggering foods in both groups of infants in our study, and with no difference between groups, were cow’s milk and egg. Koksal and colleagues found that infants with FPIAP developed tolerance to egg later than those achieving tolerance to milk [34]. Avoidance of dairy foods in the mothers’ diet did not influence the acquisition of overall tolerance, while the elimination of more foods appears to be associated with a later development of tolerance. Late tolerance development in earlier studies was reported to be associated with multiple FA [20,35].

Erdem and colleagues, compared cases that developed tolerance before and after the age of 12 months and reported earlier tolerance acquisition in those cases in which the symptoms had started at a younger age [21]. In our study, there was no significant difference in the age at diagnosis of infants who achieved resolution before and after 6 months of age.

Senocak and colleagues reported that the presence of diarrhea leads to late development of tolerance [25], a finding not confirmed in our patients.

Galip and colleagues reported that neither the skin prick test nor specific IgE or fecal calprotectin showed a significant relationship with tolerance acquisition in FPIAP, but considered that the atopy patch test may be useful in cases still refractory at the age of 18 months [23]. The oral challenge test remains the gold standard for assessing tolerance induction [36,37], although the appropriate timing is still under debate.

One study evaluating infants who developed tolerance before and after 18 months of age found that a family history of any allergic disease was a highly significant risk factor for late tolerance according to univariate, but not multivariate, logistic analysis [23]. In our study, allergy, in particular cow’s milk allergy, and asthma in siblings appeared to be risk factors for delay tolerance acquisition.

Meyer and colleagues proposed non-IgE-mediated gastrointestinal allergies, eczema, asthma and wheezing to be part of an allergic progression [38]. In our study, all eight infants with wheezing history showed later resolution of FPIAP, after the age of 6 months (i.e., they were all in group B). Uncuoglu and colleagues identified eczema as an independent risk factor for multiple FA in FPIAP, associated with late tolerance acquisition [39]. In our study, eczema as a comorbidity presented more frequently in group B, with a difference close to significance suggesting eczema as a potential risk factor for delay FPIAP resolution. More studies focusing on eczema as comorbidity are needed to clarify this association.

Current evidence supports maternal avoidance of dietary elements for the treatment of FPIAP [36] and pediatricians frequently recommend an overly restrictive approach when advising dietary eliminations [40]. According to our results, maternal avoidance of dairy products did not influence the age of development of tolerance, while the type of formula first used appeared to be associated with late tolerance acquisition. In line with other reports, the use of amino acid-based formula was associated with delayed tolerance [25]. Canani and colleagues demonstrated that the use of eHF in the dietary choices of those with an allergy to cow’s milk protein accelerated the achievement of tolerance compared with other options [41], but in our study it did not appear to affect the time of tolerance acquisition.

Celik and colleagues demonstrated that daily maternal consumption of yogurt and cheese during pregnancy was significantly lower in infants with atopic dermatitis than in healthy children [42]. In a previous study, yoghurt was tolerated in two thirds of unheated milk reactive patients suffering from non-IgE-mediated cow’s milk allergy [43]. Similarly, in our study, earlier resolution of FPIAP was promoted by maternal consumption of whole fat yogurt and goat/sheep cheese during pregnancy.

In accordance with other studies, our results show that early contact with allergens could induce earlier tolerance acquisition. Martin and colleagues showed that infants fed both breastmilk and formula at any point during the first 4 months were less likely to develop FPIAP than infants fed exclusively formula or exclusively breastmilk [4]. Even the presence of the infants in the kitchen during cooking appeared to exert a protective role [44].

We observed that tolerance developed at a later age in infants whose mothers consumed grilled food, “ready-to-eat” meals outside the house, pastries, meat and alcohol. It is possible that a maternal diet rich in these foods may cause increased intestinal inflammation in their infants, delaying the development of tolerance. In addition, group B mothers consumed multivitamins during pregnancy more frequently than group A mothers, suggesting that either the multivitamins promote a delay in the resolution of FPIAP or that taking multivitamins indicates that these mothers understand that they do not have balanced diet (including eating junk food). Conversely, other studies have reported that maternal vitamin intake offers protection against allergy outcomes in the offspring [45,46].

In Greece, as in other countries, lactating women are often advised to avoid vegetables that may cause colic in the baby [47]. Increased consumption of certain vegetables provides insoluble fiber that can increase gut motility, causing incomplete digestion and permeability of the gastrointestinal mucosa to allergenic proteins [48]. Vegetables may thus act as a co-factor in the activation of allergic reactions, similar to co-factors in IgE-mediated FA [49] or anaphylaxis [50,51]. Consumption of alkaline boiled vegetables, such as cabbage, onion and broccoli, could increase stomach pH. Low gastric pH is crucial for the degradation of proteins into small peptides, which are either ignored by the immune system, or lead to tolerance [52].

Karatas and colleagues reported that a higher maternal educational level was associated with an increased risk of FPIAP [53]. There is documentation of an association between lower parenteral education and socioeconomic status, with a poorer quality of diet and an increased risk of asthma, along with low adherence to asthma medication use being observed [54]. In our, study, once FPIAP was diagnosed, a higher maternal educational level was associated with earlier resolution of symptoms, as group A mothers more often had a higher level of education.

Parental smoking has been shown to be a significant risk factor for developing FA [44,55]. In infant monkeys, perinatal tobacco smoke exposure can induce a Th2-biased inflammatory response [56]. Only children with a family history of atopic diseases who were exposed to tobacco smoke showed an increased risk of developing an allergy [57]. The role of exposure to tobacco smoke in pregnancy and postnatally in the development of tolerance in FPIAP had not been previously studied, but we observed that maternal smoking was significantly related to later symptoms resolution.

The limitations of our study were the retrospective design and use of self-reported questionnaires for mothers to report their eating habits during pregnancy and lactation, which may entail recall bias. In some cases, tolerance may have been achieved earlier than the time of food challenge. The number of participants in each group, however, was sufficient to derive significant outcomes, which need to be confirmed with prospective, interventional studies.

## 5. Conclusions

Based on our results, mothers who have had a child with FPIAP due to cow’s milk protein could be advised to increase their consumption of traditional yogurt and goat/sheep cheese in subsequent pregnancies and to avoid consumption of multivitamins, grilled food, “ready-to-eat” meals outside the house, pastries, meat and alcohol, and to avoid smoking, in order to reduce the duration of symptoms in the case of FPIAP diagnosis in a later child. Reducing the period of FPIAP symptoms in this way provides a strategy for avoidance of prolonged exclusion diets.

Further prospective intervention studies based on these dietary changes will shed light on their effectiveness in earlier FPIAP resolution in infants, but also on their potential to decrease the risk of asthma and wheeze in this population later in life.

## Figures and Tables

**Table 1:** Characteristics of study infants with food protein-induced allergic proctocolitis (FPIAP), according to age of tolerance acquisition: Group A, before 6 months, Group B, after 6 months.

	Group A (N = 43)	Group B (N = 53)	*p*-Value
Current Age (months)	13 (9–17)	21 (14–30)	0.001
Male Sex	20 (46.51%)	28 (52.83%)	0.68
Age at Diagnosis (months)	1.5 (1–3)	1.5 (1–3)	0.57
Weight (kg) at Diagnosis	3.85 (3.725–5.03)	4.25 (3.7–5.15)	0.68
Length (cm) at Diagnosis	54.5 (52–59.875)	55 (54–61.25)	0.59
**Symptoms**			
Blood in stools	40 (93.02%)	49 (92.45%)	>0.05
Mucus in stools	39 (90.7%)	48 (90.57%)	>0.05
Anemia	0 (0%)	3 (5.66%)	0.32
Hypoalbuminemia	0 (0%)	1 (1.89%)	>0.05
Other	4 (9.3%)	5 (9.43%)	>0.05

Values are actual values (%) or mean value (range). Statistical significance *p* ≤ 0.05.

**Table 2:** Family history of atopy/asthma and demographic features of study infants with food protein-induced allergic proctocolitis (FPIAP), according to age of tolerance acquisition: Group A, before 6 months, Group B, after 6 months.

	Group A (N = 43)	Group B (N = 53)	*p*-Value
**Maternal profile**			
Milk Allergy	0 (0%)	4 (7.55%)	>0.05
Egg Allergy	0 (0%)	4 (7.55%)	>0.05
Fish Allergy	0 (0%)	1 (1.89%)	>0.05
Wheat Allergy	0 (0%)	1 (1.89%)	>0.05
Shellfish Allergy	0 (0%)	1 (1.89%)	>0.05
Peanut Allergy	0 (0%)	2 (3.77%)	>0.05
Hazelnut Allergy	0 (0%)	2 (3.77%)	>0.05
Walnut Allergy	0 (0%)	1 (1.89%)	>0.05
Sesame Allergy	0 (0%)	1 (1.89%)	>0.05
Banana Allergy	0 (0%)	1 (1.89%)	>0.05
Chocolate Allergy	0 (0%)	1 (1.89%)	>0.05
Allergic Rhinitis	0 (0%)	1 (1.89%)	>0.05
Asthma Wheeze	0 (0%)	1 (1.89%)	>0.05
Eczema	0 (0%)	1 (1.89%)	>0.05
*Smoking*	*2 (4.65%)*	*11 (20.75%)*	*0.04*
*Rural Residence*	*1 (2.33%)*	*14 (26.42%)*	*0.003*
*Secondary level of Education*	*5 (4–5)*	*2 (2–2)*	*<0.001*
**Paternal Profile**			
Milk Allergy	3 (6.98%)	7 (13.21%)	>0.05
Fish Allergy	0 (0%)	5 (9.43%)	>0.05
Wheat Allergy	1 (2.33%)	2 (3.77%)	>0.05
Peanut Allergy	0 (0%)	1 (1.89%)	>0.05
Hazelnut Allergy	0 (0%)	3 (5.66%)	>0.05
Walnut Allergy	0 (0%)	1 (1.89%)	>0.05
Sesame Allergy	0 (0%)	2 (3.77%)	>0.05
Banana Allergy	0 (0%)	2 (3.77%)	>0.05
Asthma Wheeze	1 (2.33%)	5 (9.43%)	>0.05
Eczema	7 (16.28%)	9 (16.98%)	>0.05
Smoking	3 (11.11%)	10 (28.57%)	>0.05
Residence	4 (9.3%)	8 (15.09%)	>0.05
Education	5 (5–5)	5 (5–5.75)	>0.05
**Siblings’ profile**			
*Cow’s milk Allergy*	*1 (2.33%)*	*9 (16.98%)*	*0.04*
Egg Allergy	0 (0%)	5 (9.43%)	>0.05
Fish Allergy	0 (0%)	1 (1.89%)	>0.05
Wheat Allergy	0 (0%)	2 (3.77%)	>0.05
Seafood Allergy	0 (0%)	2 (3.77%)	>0.05
Peanut Allergy	0 (0%)	1 (1.89%)	>0.05
Hazelnut Allergy	0 (0%)	1 (1.89%)	>0.05
Walnut Allergy	0 (0%)	1 (1.89%)	>0.05
Pepper allergy	0 (0%)	1 (1.89%)	>0.05
Asthma Wheeze	1 (2.33%)	8 (15.09%)	>0.05
FPIAP	3 (6.98%)	2 (3.77%)	>0.05
Eczema	2 (4.65%)	10 (18.87%)	>0.05

Values are actual value (%). Statistical significance *p* ≤ 0.05.

**Table 3:** Significant maternal dietary habits during pregnancy of study infants with food protein-induced allergic proctocolitis (FPIAP), according to time of tolerance development: Group A, before 6 months, Group B, after 6 months.

	Group A (N = 43)	Group B (N = 53)	*p*-Value
*Potatoes fried*	*1 (0–1.5)*	*1 (1–2)*	*0.04*
*Fermented Vegetables*	*0 (0–0)*	*0 (0–1)*	*0.02*
Whole Fat Yogurt	4 (0–5)	2 (0–4)	0.03
Turkey	2 (2–3)	2 (2–2)	0.02
Chicken	0 (0–0)	0 (0–1)	0.04
Pork	0 (0–1)	1 (0–2)	0.04
Beer	0 (0–0)	0 (0–1)	0.03
Olive Oil	5 (5–5)	5 (4–5)	<0.001
Seed Oil	0 (0–0)	0 (0–2)	0.02
Margarine	0 (0–0)	0 (0–2)	0.004
Butter	0 (0–2)	2 (0–2)	0.007
Yellow Cheese	2 (1.8–2.7)	1.8 (1.4–2.2)	0.007
Pastries	1.12 (0.62–1.5)	1.315 (0.75–2.12)	0.04
Grilled Food	1 (1–1)	1 (1–2)	0.01
Table Salt	3 (2–3)	2 (2–3)	0.01
“Ready-to-eat” meals	2 (2–2)	2 (2–3)	0.04
Multivitamins	8 (18.6%)	23 (43.4%)	0.01

Values are Median (Q1–Q3). Statistical significance *p* ≤ 0.05.

**Table 4:** Significant maternal dietary habits during breastfeeding of study infants with food protein-induced allergic proctocolitis (FPIAP), according to age of tolerance acquisition: Group A, before 6 months, Group B, after 6 months.

	Group A (N = 43)	Group B (N = 53)	*p*-Value
Olives	1 (0–2)	2 (0–3)	0.03
Boiled vegetables (e.g., broccoli cauliflower, zucchini)	1 (0–2)	2 (2–3)	0.03
Fermented Vegetables	0 (0–0)	0 (0–1)	0.003
Green Vegetables	1.4 (1.2–2)	2 (1.2–2.55)	0.04
Orange	1.71 (1.14–2.29)	2.5 (1.465–3.43)	0.006
Other Winter fruit	0.5 (0–3)	3 (1–4)	0.006
Olive Oil	5 (5–5)	5 (4–5)	<0.001
Margarine	0 (0–0)	0 (0–2)	0.001
Butter	0 (0–1)	1 (0–2)	0.008
Cheese	2 (1.55–2.6)	1.5 (0.6–2.2)	0.04
Red meat	0.73 (0.45–0.91)	0.82 (0.55–1.09)	0.03
White meat	1 (0.67–1.33)	1 (1–1.67)	0.02
Pastries	0.88 (0.5–1.1525)	1.12 (0.62–2.12)	0.01
Alcohol	0 (0–0)	0 (0–1)	0.03

Values are Median (Q1–Q3) Statistical significance *p* ≤ 0.05.

## Data Availability

Data supporting results can be provided upon request to the corresponding author.

## Supplementary Materials

The following supporting information can be downloaded at: https://www.mdpi.com/article/10.3390/nu15020425/s1, Table S1: Trigger foods of food protein-induced allergic proctocolitis (FPIAP), among the two group of infants, categorized according to the tolerance acquisition: Group A before 6 months, Group B after 6 months; Table S2: Maternal avoidance diet and/or milk formula selection upon FPIAP; Table S3: Reported allergy or asthma/wheeze symptoms of study population, apart from FPIAP.
