# Supplementary material for: Dietary Factors May Delay Tolerance Acquisition in Food Protein-Induced Allergic Proctocolitis
Source: Nutrients. 2023 Jan 13;15(2):425. doi: 10.3390/nu15020425 (PMC9862452; doi:10.3390/nu15020425)
Supplement: Supplementary file 1 [file nutrients-15-00425-s001.zip › nutrients-2102425-supplementary.pdf]

Supplementary Materials

Additional explanatory text for the Univariate analysis:

Following univariate analysis, the XGBoost classifier incorporated 29 features (those with $p<0.05$ during the hypothesis testing procedure) to predict the time of tolerance acquisition in months $0\leq \text{time}<6$ ή $\text{time}\geq 6$ or group A vs. group B). As a result, there was a success of 100% (96/96) during training, regarding the correct prediction of subjects, with an accuracy of 74% (71/96) during testing, i.e., during the 6-fold cross validation, and 79.2% sensitivity, 67.4% specificity, 77.1% F1-score, and 72.7% ROC area under the curve (AUC). Since hyperparameter tuning was used, optimal hyperparameter values were regarding the learning rate = 0.05, the gamma = 1, the maximum depth = 4, the minimum child weight = 1, the subsample ratio instance = 1, and the subsample ratio columns per tree = 1. The testing outcome is visualized in Figure 1, using the ROC curve achieved by the classifier.

To interpret the contribution of each predictor individually, the SHAP diagrams were illustrated, both for training (Figure 2) and the 6 folds of testing (Figure 3). The predictors included in a SHAP diagram are only those that achieve a mean SHAP > 0.1.

Among the parameters included in the model, earlier resolution of the FPIAP was always favored by a higher level of maternal education and higher consumption of goat/sheep cheese during pregnancy. Salt intake during pregnancy and olive oil during breastfeeding were correlated with earlier resolution. Conversely, consumption of multivitamins during pregnancy and intake of white and red meat, winter fruits, green vegetables, butter, salt,

“ready-to-eat” meals and pastries during breastfeeding were correlated with longer duration of symptoms.

Table S1

Trigger foods of food protein-induced allergic proctocolitis (FPIAP), among the two group of infants, categorized according to the tolerance acquisition: Group A before 6 months, Group B after 6 months

	**Group A (N=43)**	**Group B (N=53)**	**p-value**
**Trigger food**			
Milk	42 (97.67%)	50 (94.34%)	0.76
Egg	1 (2.33%)	7 (13.21%)	0.12
Wheat	0 (0%)	6 (11.32%)	0.06
Corn	0 (0%)	2 (3.77%)	0.57
Soya	0 (0%)	3 (5.66%)	0.32
Beef	1 (2.33%)	6 (11.32%)	0.19
Lamp	0 (0%)	1 (1.89%)	1
Peanut	0 (0%)	1 (1.89%)	1
Hazelnut	0 (0%)	0 (0%)	1
Walnut	0 (0%)	0 (0%)	1
Fish	0 (0%)	1 (1.89%)	1
Rice flour	1 (2.33%)	0 (0%)	0.91
Pear	0 (0%)	1 (1.89%)	1
Grape	0 (0%)	1 (1.89%)	1

Values are actual values (%) or mean value (range)

Statistical significance $p \leq 0.05$

Table S2: Maternal avoidance diet and /or milk formula selection upon FPIAP

	Group A (N=43)	Group B (N=53)	p-value
**Maternal avoidance diet**			
Maternal avoidance of dairy products	29 (67.44%)	31 (58.49%)	0.49
Maternal avoidance of other foods	*0 (0%)*	*11 (20.75%)*	*0.004*
Exclusive Breastfeeding	5 (3 - 7)	5 (3 - 6)	0.64
**Milk formula selection**			
Age of first formula introduction (months)	*0 (0 - 3)*	*3 (1 - 5.5)*	*0.001*
Ext Hydr	10 (23.26%)	12 (22.64%)	1
Amino Acid	0 (0%)	5 (9.43%)	0.11
Cow's milk	14 (32.56%)	18 (33.96%)	1
HA	5 (11.63%)	10 (18.87%)	0.49
Elemental	*2 (4.65%)*	*14 (26.42%)*	*0.01*
Probiotics	5 (11.63%)	9 (16.98%)	0.65

Values are actual values (%) or median (Q1-Q3)

Statistical significance $p \leq 0.05$

Table S3: Reported allergy or asthma/wheeze symptoms of study population, apart from FPIAP

Milk Allergy	28 (65.12%)	29 (54.72%)	0.41
Egg Allergy	4 (9.3%)	4 (7.55%)	1
Fish Allergy	0 (0%)	0 (0%)	1
Wheat Allergy	0 (0%)	4 (7.55%)	0.18
Shellfish Allergy	0 (0%)	0 (0%)	1
Peanut Allergy	0 (0%)	1 (1.89%)	1
Soy Allergy	0 (0%)	1 (1.89%)	1
Beef Allergy	1 (2.33%)	4 (7.55%)	0.49
Lamp Allergy	0 (0%)	1 (1.89%)	1
Hazelnut Allergy	1 (2.33%)	1 (1.89%)	1
Walnut Allergy	0 (0%)	0 (0%)	1
Chicken Allergy	0 (0%)	1 (1.89%)	1
Apple Allergy	0 (0%)	1 (1.89%)	1
Carrot Allergy	0 (0%)	1 (1.89%)	1
Goat sheep Allergy	0 (0%)	1 (1.89%)	1
Asthma/Wheeze	*0 (0%)*	*8 (15.09%)*	*0.02*
Eczema	5 (11.63%)	16 (30.19%)	0.05

Values are actual values (%)

Statistical significance $p \leq 0.05$
